# Understanding diagnostic pathways in systemic sclerosis and systemic sclerosis-associated interstitial lung disease: A retrospective cohort study

**DOI:** 10.1097/MD.0000000000029993

**Published:** 2022-08-12

**Authors:** Shervin Assassi, Nan Shao, Ziwei Yin, Elizabeth R. Volkmann, Donald F. Zoz, Thomas B. Leonard

**Affiliations:** a Division of Rheumatology, University of Texas McGovern Medical School, Houston, TX, USA; b Boehringer Ingelheim Pharmaceuticals, Inc., Ridgefield, CT, USA; c Department of Medicine, Division of Rheumatology, University of California, David Geffen School of Medicine, Los Angeles, CA, USA.

**Keywords:** administrative claims, delayed diagnosis, healthcare, pulmonary fibrosis, scleroderma, systemic

## Abstract

Systemic sclerosis-associated interstitial lung disease (SSc-ILD) is usually detected in a patient known to have SSc but may be diagnosed prior to SSc. We probed an insurance database to investigate documentation of ILD prior to SSc. Using Optum’s Clinformatics^®^ Data Mart Database, we identified patients with an SSc index date between January 1, 2010, and September 30, 2015, based on International Classification of Diseases (ICD)-9-Clinical Modification (CM) codes, ≥2 medical claims associated with SSc on different dates within 1 year, and ≥3 years of continuous enrollment prior to SSc index date (ICD-9-CM cohort). We identified an ICD-10-CM cohort comprising patients with an SSc index date between October 1, 2017, and June 30, 2019, based on ICD-10-CM codes, ≥2 medical claims associated with SSc on different dates within 1 year, and ≥2 years of continuous enrollment prior to SSc index date. ILD was defined as ≥2 medical claims associated with ILD on different dates. The ICD-9-CM and ICD-10-CM cohorts comprised 1779 and 1032 patients, respectively. In these cohorts, respectively, 7.6% and 9.3% of patients had their second medical claim associated with ILD prior to their SSc index date, and 4.3% and 5.6% of patients had their second medical claim associated with ILD >1 year prior to the SSc index date. In this analysis, 4% to 6% of patients with SSc had claims for ILD >1 year prior to a claim for SSc. These data show that SSc can affect the lung early and demonstrate the importance of screening patients with SSc for ILD and patients with ILD for SSc.

## 1. Introduction

Systemic sclerosis (SSc) is a heterogeneous connective tissue disease (CTD) characterized by progressive fibrosis of the skin and internal organs.^[1]^ Interstitial lung disease (ILD) is a common manifestation of SSc and the leading cause of SSc-related death.^[2,3]^ The reported prevalence of ILD among patients with SSc varies depending on the population studied and the methodology used. Among patients with SSc in the Canadian Scleroderma Research Group registry, 52% of patients (and 64% of 289 patients with high-resolution computed tomography [HRCT] scans) were diagnosed with ILD.^[4]^ Among patients with SSc in the European Scleroderma Trials and Research database, 35% of 8013 patients with limited cutaneous SSc and 57% of 4786 patients with diffuse cutaneous SSc had ILD on HRCT or X-ray.^[5]^ A recent analysis of US claims data estimated the prevalence of SSc to be 24.4 per 100,000 and the prevalence of SSc-ILD to be 6.9 per 100,000.^[6]^

CTD-ILD is usually detected in a patient who is already known to have a CTD, but patients initially diagnosed with idiopathic ILD may be diagnosed with a CTD-ILD after further assessment.^[7–10]^ In some patients with SSc, pulmonary manifestations precede extrapulmonary manifestations by several months or even years.^[7,8,11–13]^ To gain a better understanding of diagnostic pathways in patients with SSc/SSc-ILD, we used data from a large US insurance claims database to investigate the documentation of ILD prior to a diagnosis of SSc.

## 2. Methods

We used data from Optum’s de-identified Clinformatics^®^ Data Mart Database, a commercial and Medicare Advantage claims database that covers >60 million patients in the United States. Data came from the period January 1, 2007, to June 30, 2019. Two cohorts of patients with SSc aged ≥18 years were defined based on codes in the ninth and tenth revisions of the International Classification of Diseases, Clinical Modification (ICD-9-CM and ICD-10-CM). These cohorts were selected to cover the periods before and after the transition from ICD-9-CM to ICD-10-CM on October 1, 2015. The ICD-9-CM cohort included individuals with an SSc index date (first insurance claim related to SSc) between January 1, 2010, and September 30, 2015, based on ICD-9-CM codes, ≥2 medical claims related to SSc on different dates within 1 year, and ≥3 years continuous enrollment prior to their index date. The ICD-10-CM cohort included individuals with an SSc index date between October 1, 2017, and June 30, 2019, based on ICD-10-CM codes, ≥2 medical claims related to SSc on different dates within 1 year, and ≥2 years continuous enrollment prior to their index date. ILD was defined as ≥2 claims related to ILD on different days. ICD-9-CM and ICD-10-CM codes for SSc and ILD are summarized in Tables 1 and 2 (Supplemental Digital Content, http://links.lww.com/MD/G990). For the ICD-9-CM cohort, we selected a period of ≥3 years continuous enrollment in the database prior to the SSc index date to ensure we collected enough data to enable a robust analysis. Due to a shorter length of enrollment in the ICD-10-CM cohort, in this cohort, a period of ≥2 years of continuous enrollment in the database prior to the SSc index date was chosen.

In descriptive analyses, we determined the proportion of patients who had their second claim associated with ILD prior to their SSc index date (see Table 1 for definitions). We then compared the characteristics of patients who had their second claim associated with ILD >1 versus ≤1 year prior to their SSc index date based on pooled data from the ICD-9-CM and ICD-10-CM cohorts. Among patients who had ILD >1 year prior to their SSc index date in the ICD-9-CM cohort, we assessed the most frequently seen providers and most frequently conducted procedures related to a patient’s first or second ILD claim. Provider codes were preprocessed to remove duplicates. A patient was counted in multiple categories if they saw providers of different types or if multiple procedures were conducted. Procedure codes related to “office visits” were excluded as they were deemed too general. Lastly, in a subgroup of patients in the ICD-9-CM cohort who had ≥3 years of continuous enrollment in the database both prior to and following the SSc index date, we compared the characteristics of patients who had their second claim associated with ILD prior to their SSc index date with those who had their first claim associated with ILD after their SSc index date.

**Table 1:** Definitions of SSc, ILD, and ILD prior to SSc.

Diagnosis	Definition
SSc	≥2 medical claims related to SSc
ILD	≥2 medical claims related to ILD
ILD prior to SSc	Second medical claim for ILD prior to first medical claim for SSc (i.e., the index date)

We also investigated the documentation of ILD prior to a diagnosis of SSc using data from the de-identified US-based Cerner Health Facts^®^ database. Unlike data from Optum, which are based on claim-level data, data from Cerner are based on encounter-level data extracted from electronic medical records. The Cerner database contains information from approximately 69 million US patients. The study period for the Cerner ICD-9-CM cohort was from January 1, 2000, to September 30, 2018. Based on ICD-9-CM codes, we identified patients with ≥2 medical encounters with SSc diagnosis codes on different dates within a 1-year period, and ≥3 years of medical history prior to their first SSc encounter.

As this analysis included de-identified data from the Optum Clinformatics Data Mart database and the Cerner Health Facts database, approval from an ethics committee or institutional review board was not required.

## 3. Results

### 3.1. Patients

Using the Optum database, based on ICD-9-CM codes, we identified 13,477 patients with ≥2 medical claims associated with SSc on different dates within a 1-year period; of these patients, 1779 patients had ≥3 years of continuous enrollment prior to the SSc index date and comprised the Optum ICD-9-CM cohort. Based on ICD-10-CM codes, we identified 11,783 patients with ≥2 medical claims associated with SSc on different dates within a 1-year period; of these patients, 1032 had their SSc index date between October 1, 2017, and June 30, 2019, and had ≥2 years continuous enrollment prior to the SSc index date and comprised the Optum ICD-10-CM cohort. Thirteen patients (0.5%) were counted in both the Optum ICD-9-CM and ICD-10-CM cohorts.

At the time of their first SSc claim, patients in the Optum ICD-9-CM cohort had a mean (standard deviation [SD]) age of 60.8 (14.2) years; 81.6% were female, and 65.5%, 13.8%, 10.8%, and 2.9% were White, Hispanic, Black, and Asian, respectively. Patients in the Optum ICD-10-CM cohort had a mean (SD) age of 65.0 (13.7) years at the time of their first claim; 82.8% were female, and 64.5%, 12.0%, 9.1%, and 4.3% were White, Hispanic, Black, and Asian, respectively.

Based on the Cerner database, we identified 4969 patients with ≥2 medical encounters with SSc diagnosis codes on different dates within a 1-year period. Of these, 476 patients had ≥3 years of medical history prior to their first SSc encounter and comprised the Cerner ICD-9-CM cohort. Patients in the Cerner ICD-9-CM cohort had a mean (SD) age of 58.4 (14.5) years at the time of their first SSc diagnosis; 91.8% were female, and 75.0%, 17.7%, 0.8%, and 0.6% were White, African American, Hispanic, and Asian, respectively.

### 3.2. Patients who had their second ILD claim prior to their SSc index date

In the Optum ICD-9-CM and ICD-10-CM cohorts, respectively, 136 patients (7.6%) and 96 patients (9.3%) had their second medical claim related to ILD prior to their SSc index date, 76 patients (4.3%) and 58 patients (5.6%) had their second medical claim related to ILD >1 year prior to the SSc index date (Table 2). In the Cerner ICD-9-CM cohort, 16 patients (3.4%) had their second ILD encounter >1 year prior to their first SSc encounter.

**Table 2:** Patients who had second ILD claim prior to SSc index date in Optum database.

	ICD-9-CM cohort (n = 1779)	ICD-10-CM cohort (n = 1032)
ILD at any time prior to SSc	136 (7.6)	96 (9.3)
ILD >90 d prior to SSc	100 (5.6)	76 (7.4)
ILD >180 d prior to SSc	92 (5.2)	68 (6.6)
ILD >1 yr prior to SSc	76 (4.3)	58 (5.6)
ILD >2 yr prior to SSc	48 (2.7)	36 (3.5)

**Figure 1.**
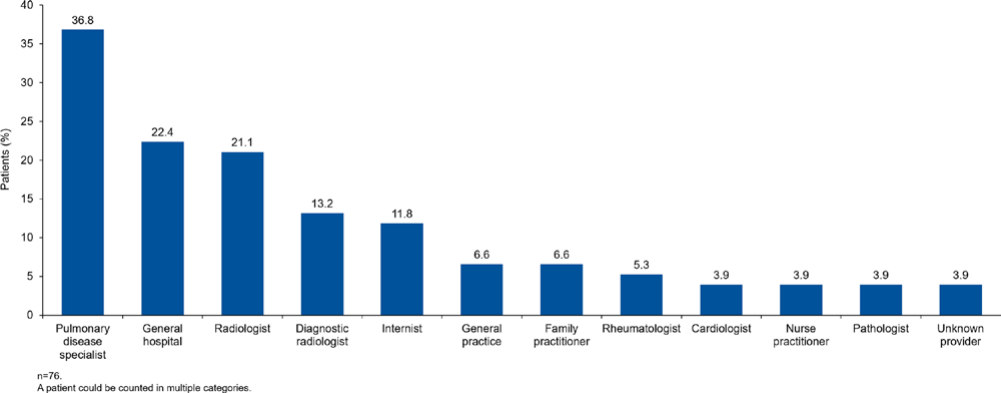
Providers who most commonly submitted claims during first or second ILD claim by patients in the Optum ICD-9-CM cohort with second ILD claim >1 yr prior to SSc index date. ICD-CM = International Classification of Diseases, Clinical Modification, ILD = interstitial lung disease, SSc = systemic sclerosis.

**Figure 2.**
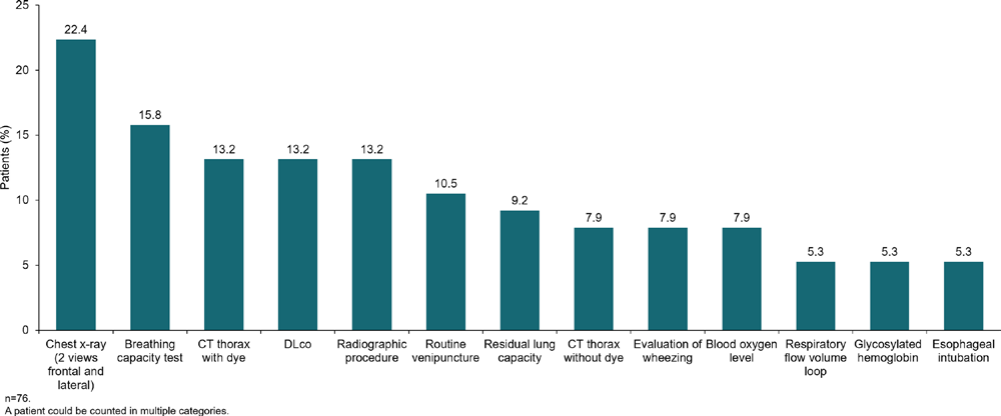
Procedures conducted during first or second ILD claim by patients in the Optum ICD-9-CM cohort with second ILD claim >1 yr prior to SSc index date. CT = computed tomography, DLco, diffusing capacity of the lung for carbon monoxide, ICD-CM = International Classification of Diseases, Clinical Modification, ILD = interstitial lung disease.

Based on pooled data from the Optum ICD-9-CM and ICD-10-CM cohorts, there were no significant differences in the demographic characteristics of patients who had their second medical claim associated with ILD >1 year versus ≤1 year prior to their SSc index date (Table 3). Of the 76 patients in the Optum ICD-9-CM cohort who had their second medical claim associated with ILD >1 year prior to their SSc index date, 5.3% were aged 18 to 40 years, 9.2% were aged 41 to 50 years, 23.7% were aged 51 to 60 years, 30.3% were aged 61 to 70 years, 25.0% were aged 71 to 80 years, and 6.6% were aged >81 years. In these patients, the providers who most commonly submitted claims during the first or second ILD claim were pulmonary disease specialists, general hospital providers, and radiologists (Fig. 1). The most commonly conducted procedures were chest x-ray, breathing capacity tests, and radiographic procedures (Fig. 2).

**Table 3:** Characteristics of patients who had their second medical claim associated with ILD >1 yr versus ≤1 yr prior to their SSc index date (Optum ICD-9-CM and ICD-10-CM cohorts combined).

	ILD at any time prior to SSc (n = 232)	ILD >1 yr prior to SSc (n = 134)	ILD ≤1 yr prior to SSc (n = 98)	*P* value*
Age, yr, mean (SD)	66.0 (12.2)	65.5 (11.9)	66.7 (12.6)	.47
Female, n (%)	182 (78.4)	111 (82.8)	71 (72.4)	.06
Race, n (%)				1.00
White	152 (65.5)	88 (65.7)	64 (65.3)	
Black	38 (16.4)	22 (16.4)	16 (16.3)	
Hispanic	26 (11.2)	15 (11.2)	11 (11.2)	
Asian	4 (1.7)	2 (1.5)	2 (2.0)	
Missing	12 (5.2)	7 (5.2)	5 (5.1)	

In total, 923 patients in the Optum ICD-9-CM cohort had ≥3 years of continuous enrollment in the database both prior to and following the SSc index date, of whom 200 (21.6%) had a claim associated with ILD. There were no significant differences in the demographic characteristics of patients who had their second claim associated with ILD prior to their SSc index date (n = 52) and those who had their first claim associated with ILD after their index date (n = 127; Table 4).

**Table 4:** Characteristics of patients who had their second claim associated with ILD prior to their SSc index date and who had their first claim associated with ILD after their SSc index date in the Optum ICD-9-CM cohort.

	Second claim associated with ILD prior to SSc index date (n = 52)	First claim associated with ILD after SSc index date (n = 127)	*P* value*
Age, yr, mean (SD)	62.6 (13.0)	60.9 (12.1)	.46
Female, n (%)	43 (82.7)	98 (77.2)	.30
Race, n (%)			.55
White	29 (55.8)	73 (57.5)	
Black	9 (17.3)	14 (11.0)	
Hispanic	10 (19.2)	18 (14.2)	
Asian	2 (3.9)	9 (7.1)	
Missing	2 (3.9)	13 (10.2)	

## 4. Discussion

We used data from a large US insurance claims database to investigate the documentation of ILD prior to a diagnosis of SSc. In this database, 4% to 6% of patients with SSc had claims for ILD >1 year prior to a claim for SSc. These findings were supported by analyses of a database based on electronic medical records, which showed that 3.4% of patients had a second ILD encounter >1 year prior to their first SSc encounter. Based on the Optum database, we found no differences in the demographic characteristics of patients who had claims for ILD prior to versus after a claim for SSc or claims for ILD >1 year versus <1 year prior to a claim for SSc.

These data support previous studies showing that SSc can damage the lungs early in the course of the disease.^[13–17]^ Expert groups have proposed that all patients diagnosed with SSc should be screened for ILD, including using HRCT, which is the gold standard methodology for detection of ILD.^[18–21]^ Our findings also underline the importance of clinical examination and serologic testing in patients presenting with an ILD to exclude autoimmune disease as the underlying cause, as recommended in international diagnostic guidelines.^[22–24]^ Progression of SSc-ILD is associated with a significant increase in mortality.^[25,26]^ Early identification of SSc-ILD is important to enable patients to receive prompt treatment to slow progression and improve outcomes.^[19]^ Two drugs—nintedanib and tocilizumab—have been approved by the Food and Drug Administration for slowing decline in lung function in patients with SSc-ILD. Randomized placebo-controlled trials have shown that these therapies slow the decline in forced vital capacity in patients with SSc-ILD.^[27,28]^ Recently published international guidelines for the management of progressive pulmonary fibrosis provided a conditional recommendation for the use of nintedanib in patients with progressive pulmonary fibrosis who have failed standard management.^[29]^

Previous studies have shown that a proportion of patients who present with an ILD and no symptoms of a CTD have a CTD on further evaluation. In a retrospective cohort of 114 patients referred to an ILD clinic at a tertiary care center, 17 (14.9%) were newly diagnosed with CTD as a direct consequence of their evaluation at the ILD clinic.^[7]^ A study of 50 patients with ILD evaluated at a tertiary referral center found that of the 25 patients who had a final diagnosis of CTD-ILD, 7 (28%) had been referred with a diagnosis of idiopathic pulmonary fibrosis.^[9]^ Similarly, in a prospective study of 60 patients with newly diagnosed ILD, 6 (21%) of 28 patients with an initial diagnosis of idiopathic pulmonary fibrosis had their diagnosis changed to a CTD-ILD after rheumatological tests.^[10]^ It is also clear that some patients are significantly delayed in receiving a diagnosis of SSc. Among 221 consecutive patients with SSc assessed at a Spanish center, the mean time from onset of symptoms to diagnosis of SSc was 6.3 years.^[30]^ In a survey of 64 patients with SSc, 21% of patients were diagnosed with SSc >4 years after the onset of symptoms.^[31]^ In an analysis of primary care records of 854 patients who had codes for Raynaud’s phenomenon recorded prior to codes for SSc, the period between these codes being recorded was >10 years in 22.4% of patients.^[32]^ Delays in diagnosis are likely attributable to a lack of awareness of the early symptoms of SSc among primary care physicians and to patients not consulting a physician for early symptoms of SSc such as Raynaud’s phenomenon and fatigue.^[33]^

In our study, greater proportions of patients had a second ILD claim prior to the SSc index date when SSc and ILD were based on ICD-10-CM codes compared with ICD-9-CM codes. The ICD-10-CM coding system was developed to comprise a greater number of diagnostic and procedural codes than ICD-9-CM, improving the documentation of specific diseases.^[34–36]^ Thus, the mandated introduction of the ICD-10-CM coding system in October 2015 may have led to improved capture of patients who had claims for ILD prior to claims for SSc.

Strengths of our analyses include the use of a large health insurance database that is broadly representative of the US population. The consistency of the analyses based on ICD-9-CM codes and ICD-10-CM codes, and the similar findings of analyses based on an independent database based on electronic medical records, improves confidence in our findings. A limitation of our analysis is that SSc and ILD were defined based on administrative data, so patients who did not have an ICD-9-CM or ICD-10-CM code for SSc or ILD were not included. Thirteen patients were counted in both the Optum ICD-9-CM cohort and ICD-10-CM cohorts; however, these patients comprised <1% of the cohort and there was no overlap between patients who had ILD >1 year prior to a claim for SSc in the ICD-9-CM cohort and ICD-10-CM cohorts. Further, the results of each ILD-related procedure were not available. Data on access to a specialized SSc center or on patients’ clinical characteristics that might be associated with a delay in receiving a diagnosis of SSc were not collected.

## 5. Conclusions

In conclusion, analyses of a large US health insurance database showed that 4% to 6% of patients with SSc had claims for ILD >1 year prior to a claim for SSc. These data provide further evidence that SSc can affect the lung at an early stage of SSc and reinforce the importance of screening patients with SSc for ILD and patients with ILD for SSc.

## Acknowledgments

The authors meet criteria for authorship as recommended by the International Committee of Medical Journal Editors. The authors were not paid for development of this article. Writing support was provided by Julie Fleming and Wendy Morris of Fleishman-Hillard, London, UK, funded by Boehringer Ingelheim Pharmaceuticals, Inc. Boehringer Ingelheim reviewed the article for medical and scientific accuracy and for intellectual property considerations.

## Author contributions

Conceptualization: NS, ZY, DFZ, TBL.

Formal analysis: NS and ZY.

Interpretation of the data: all authors.

Writing (editing and review): all authors.

## Supplementary Material



## Footnotes

* For the comparison between patients with ILD >1 versus ≤1 yr prior to SSc. Comparisons were based on a 2-sided *t* test, a 2-sided binomial proportion test, or a Fisher exact test.

* Comparisons were based on a 2-sided *t* test, a 2-sided binomial proportion test, or a Fisher exact test.
