# Supplementary material for: Understanding diagnostic pathways in systemic sclerosis and systemic sclerosis-associated interstitial lung disease: A retrospective cohort study
Source: Medicine (Baltimore). 2022 Aug 12;101(32):e29993. doi: 10.1097/MD.0000000000029993 (PMC9371507; doi:10.1097/MD.0000000000029993)
Supplement: Supplementary file 1 [file medi-101-e29993-s001.pdf]

Supplemental material

Supplemental Table 1. ICD-9-CM codes for SSc and ILD

SSc	710.1	Systemic sclerosis
	517.2	Lung involvement in systemic sclerosis
ILD	515	Postinflammatory pulmonary fibrosis
	516.0	Pulmonary alveolar proteinosis
	516.3	Idiopathic interstitial pneumonia
	516.30	Idiopathic interstitial pneumonia, not otherwise specified
	516.31	Idiopathic pulmonary fibrosis
	516.32	Idiopathic non-specific interstitial pneumonitis
	516.33	Acute interstitial pneumonitis
	516.34	Respiratory bronchiolitis interstitial lung disease
	516.35	Idiopathic lymphoid interstitial pneumonia
	516.36	Cryptogenic organizing pneumonia
	516.37	Desquamative interstitial pneumonia
	516.8	Other specified alveolar and parietoalveolar pneumonopathies
	516.9	Unspecified alveolar and parietoalveolar pneumonopathy
	517.2	Lung involvement in systemic sclerosis
	517.8	Lung involvement in other diseases classified elsewhere

Supplemental Table 2. ICD-10-CM codes for SSc and ILD

SSc	M34	Systemic sclerosis [scleroderma]
	M34.x	M34.0, M34.1, M34.2, M34.8, M34.81, M34.82, M34.83, M34.89, M34.9
ILD	M34.81	Systemic sclerosis with lung involvement
	J84.x	J84.01, J84.09, J84.10, J84.111, J84.112, J84.113, J84.114, J84.115, J84.116, J84.117, J84.17, J84.2, J84.89, J84.9
